# Epilogue

**Published:** 1891-01-03

**Authors:** 


### EPILOGUE.

We have touched upon but a few of the living and interesting questions that have claimed the attention of the medical and scientific worlds during the past twelve months, and that still ask for solution. The great problems of the medical and surgical treatment of the sick, of the nursing and hygienic management of the sick room, of the economic and efficient administration of hospitals and their affairs, are questions that belong not to the medical profession only but to the world, and must belong to the world in an ever-increasing degree. It is for the world and the medical profession to approach those problems in the spirit of courage and hope, and with a cultivated scientific intelligence. When this is done, and only when this is done, truth reveals her face, and mankind marches steadily forward with worthier aims and to nobler ends.

